# Effect of different beverages and polishing techniques on colour stability of CAD/CAM composite restorative materials

**DOI:** 10.2340/biid.v11.40591

**Published:** 2024-04-26

**Authors:** Lippo Lassila, Mine B. Uctasli, Kanae Wada, Pekka K. Vallittu, Sufyan Garoushi

**Affiliations:** a Department of Biomaterials Science and Turku Clinical Biomaterial Center – TCBC, Institute of Dentistry, University of Turku, Finland; b Department of Restorative Dentistry, Faculty of Dentistry, University of Gazi, Turkey; c Department of Pediatric Dentistry / Special Needs Dentistry, Tokyo Medical and Dental University TMDU, Tokyo, Japan; d Wellbeing Services County of South-West Finland, Turku, Finland

**Keywords:** Colour stability, staining, SFRC CAD, e, max, polishing technique

## Abstract

**Objectives:**

The aim of this article was to compare the colour stability of short fibre-reinforced computer-assisted design/computer-assisted manufacturing (CAD/CAM) composite (SFRC CAD) to commercially available CAD/CAM materials following prolonged immersion in a variety of beverages. Furthermore, the influence of the polishing technique was evaluated.

**Materials and methods:**

A total of 120 rectangular specimens (10 mm length × 14 mm width × 2 mm thickness) were prepared from SFRC CAD, IPS e-max, Cerasmart 270, Celtra Duo, Enamic, and Brilliant Crios. The specimens underwent polishing through either a laboratory polishing machine equipped with 4000-grit silicon carbide paper or chairside polishing using Sof-Lex spiral. Twenty specimens of each tested CAD/CAM material were randomly divided into four groups (*n* = 5) based on the staining solution used in order to evaluate the colour stability of the materials. Group 1: distilled water, Group 2: coffee, Group 3: red wine, Group 4: coke. Using a spectrophotometer, the colour changes (∆E) of all CAD/CAM materials were assessed at baseline, and after 1 and 12 weeks of staining. Three-way analysis of variance was used to analyse the data (α = 0.05).

**Results:**

The staining solution and material type showed a significant influence on the CAD/CAM specimens’ colour stability (*p* < 0.05), while polishing method had no significant influence (*p* > 0.05). The average ∆E values for specimens submerged in wine were considerably higher (*p* < 0.05) than those for the other solutions. SFRC CAD, Cerasmart 270, and Enamic displayed the highest ∆E values in wine (*p* < 0.05).

**Conclusions:**

The colour stability of tested SFRC CAD was comparable to other composite-based CAD/CAM materials, while IPS e.max exhibited the highest level of colour stability.

## Introduction

Recently, there has been a remarkable increase in patients’ expectations and interest in indirect restorations. In response to these growing demands, the adoption of metal-free fixed restorations has become highly accepted, driven by their exceptional biocompatibility, chemical and colour stability, reduced surface roughness, and aesthetic advantages [1]. Simultaneously, the use of computer-assisted design/computer-assisted manufacturing (CAD/CAM) has increased owing to significant technological advancements. The primary benefit of CAD/CAM technology lies in the capacity to use uniform and flawless CAD/CAM blocks for creating aesthetic restorations in a single session [2]. Currently on the market, there are CAD/CAM block materials made of glass ceramics, zirconia, composites, and hybrid ceramics [2, 3].

Due to its mechanical and bonding characteristics, lithium disilicate glass ceramic has been used widely for indirect single unit bonded permanent prostheses. Its flexural strength, fracture resistance, and bond strength to resin composite luting cements are all improved because of the large number of longitudinal crystals it contains [3]. However, it has been noted that lithium disilicate glass ceramic without proper polishing causes wear to antagonist enamel and is a stiff, brittle material that needs more crystallisation to obtain its maximum strength [4]. Nevertheless, adhesion failure may occur as a result of the high elastic modulus, which tends to concentrate extra stress at the adhesive interface [5]. When it comes to zirconia, scientific investigations conducted in controlled laboratory settings have determined that establishing a strong and durable bond between zirconia and the luting cement can be challenging, potentially compromising its long-term durability [6]. Zirconia, especially translucent modifications, have low fracture toughness which limits use of the material clinically [7].

To achieve a more acceptable stress distribution between restoration and underlaying tooth structure, some researchers [8, 9] advocate using materials having an elastic modulus similar to that of dentin. Due to elastic modulus, wear characteristics, colour integration, and malleability in thin layers, composite-based CAD/CAM materials have become increasingly common over the past 10 years [10, 11]. Compared to traditional light-cured resin composites, CAD/CAM composites have significantly higher monomer conversion due to the high temperature and pressure during polymerisation, resulting in composites with superior mechanical properties and biocompatibility [2]. Clinical outcome of composite-based CAD/CAM materials is still debatable to some extent, though, as evidence suggests many of their mechanical characteristics can be classified as those of ceramics [12, 13].

The development of discontinuous (short) fibre-reinforced CAD/CAM composite (SFRC CAD) aims at enhancing fracture toughness, which will enhance the mechanical characteristics of these composite-based CAD/CAM materials, and reduce issues that might have a negative impact on their long-term clinical performance [14–16]. SFRC CAD had shown good performance when mechanical, loading, optical, surface, and bonding properties were investigated [13–17]. However, no information is available regarding this material’s colour stability following exposure to staining solutions. Therefore, the current study’s objective was to assess how well SFRC CAD composite maintained its colour after being exposed to common beverages, in comparison to commercially available ceramic and composite-based CAD/CAM materials. The following are the null hypotheses that this study examined: The type of beverage, immersion time, the CAD/CAM material, and the polishing technique have no impact on the staining of restorations.

## Materials and methods

A list of the CAD/CAM materials used in the investigation is given in Table 1. Twenty rectangular specimens (10 mm length × 14 mm width × 2 mm thickness) were cut with a water-cooled, low-speed diamond saw (Struers, Glasgow, Scotland) from each CAD/CAM material. Previous research in the literature served as the basis for calculating the sample size [18, 19].

**Table 1:** The utilised CAD/CAM materials.

Material (shade)	Manufacturer	Composition* (wt%)
IPS e-max CAD (HT,A2)	Ivoclar Vivadent AG, Schaan Liechtenstein	Silicon dioxide 57–80%, Lithium oxide 11–19%, Potassium oxide 0–13%, Phosphorus oxide 0–11% and other oxides
Cerasmart 270 (LT,A2)	GC Corp, Tokyo, Japan	Bis-MEPP, UDMA, dimethacrylate , Silica (20 nm), barium glass (300 nm) 71 wt%
Celtra Duo (LT,A2)	Dentsply Sirona , Germany	Zirconium-oxide 10.1%, Silicon dioxide 58%, Lithium oxide 18.5%, Phosphorus pentoxide 5%, Alumina 1.9%
Enamic (translucent)	VITA Zahnfabrik, Bad Säckingen, Germany	UDMA, TEGDMA, glass ceramic 86 wt%
Brilliant Crios (LT,A3)	Coltene, Switzerland	Bis-GMA, Bis-EMA, TEGDMA. 71 wt% barium glass
SFRC CAD (translucent)	Experimental, Stick Tech-GC member, Finland	UDMA, TEGDMA, Short glass fibre (200–300 µm & Ø7 μm), Barium glass 77 wt%

* Composition based on manufacturer details.

TEGDMA: triethylene glycol dimethacrylate; UDMA: urethane dimethacrylate; Bis-MEPP: Bis (p-methacryloxy (ethoxy)1-2 phenyl)-propane; Bis-GMA: bisphenol-A-glycidyl dimethacrylate; Bis-EMA: Ethoxylated bisphenol-A-dimethacrylate; wt%: weight percentage.

The specimens underwent polishing by rubbing them with 600-grit silicon carbide abrasive paper while water was flowing. After reaching the desired thickness, the lower surface of the specimen was polished using abrasive paper (1000-grit, 2000-grit, and 4000-grit FEPA) for 20 s each at 300 rpm, with water cooling, using an automatic grinding machine (Struers Rotopol-11, Copenhagen, Denmark). This polished side is referred to as the ‘machine polish’ side. The specimens’ upper surface was polished using 1000-grit abrasive paper. Afterwards, the surfaces underwent polishing using a 3M™ Sof-Lex™ Diamond Polishing System, specifically utilising a pre-polishing spiral and a diamond-impregnated polishing spiral. Each spiral was polished for 20 s using a slow speed hand piece while being cooled with water. This side was referred to as the hand polish. A single operator was responsible for processing all of the specimens.

To achieve consistency, a digital calliper manufactured by Mitutoyo Corp. (Tokyo, Japan) with a precision of 0.01 mm was used to measure the thickness of each specimen. After the polishing technique, the specimens were immersed in distilled water and kept at a temperature of 37°C for 24 h prior to taking the initial colour measurement.

In order to assess the colour change across four distinct beverages, a total of 20 specimens (*n* = 5 per group) from each CAD/CAM material were randomly allocated into four groups based on the staining solution employed. Group 1 consisted of distilled water with a pH of around 7. Group 2 had Nescafe classic coffee from Nestle, where 3.6 grams of coffee powder was dissolved in 300 millilitres of boiling pure water, resulting in a pH of approximately 5. Group 3 consisted of red wine (Frontera Cabernet Sauvignon, Chile) with a pH of approximately 3.5. Group 4 contained coke from the Coca-Cola Company, and had a pH of approximately 2.5.

The specimens were immersed in containers holding the staining solutions, ensuring that both sides were exposed to the solutions. In order to prevent the evaporation of staining solutions, the specimens were kept in sealed containers for the whole course of the investigation. The stained beverages were renewed on a weekly basis. The specimens were kept in an incubator at 37°C all the time, except for when colour measurements and solution changes were made.

The colour variations (∆E) of all specimens were measured at baseline, after 1 week, and after 12 weeks of staining. Following the staining period, the specimens underwent a rinsing process using distilled water, were dried using a paper towel, and then subjected to colour evaluation from both sides.

The spectrophotometer Konica Minolta VLTCM-700d (Tokyo, Japan) was utilised to measure spectral reflectance and colour at baseline, and after staining. The measurements were conducted using the CIE Lab colour scale. The spectrophotometer utilised an aperture with a diameter of 3 mm and black background. The lighting and viewing setup adhered to the CIE diffuse/80 geometry, incorporating the specular component (SCI) geometry.

In order to determine the colour difference (∆E), the average values of ∆L, ∆a, and ∆b for each specimen were employed in the subsequent formula:

ΔE = [(ΔL*)^2^+(Δa*)^2^+(Δb*)^2^]^1/2^

Where variables ∆L, ∆a, and ∆b represent the variations in the L*, a*, and b* values respectively, between the baseline and after staining.

The variable L* indicates the lightness of the colour, ranging from black to white. The variable a* provides information on the colour’s position on the green and red axis, while b* represents its position on the yellow and blue axis.

According to earlier research [18, 20, 21], a noticeable alteration in colour with a ΔE value greater than 1 will be considered acceptable, as long as it does not exceed ΔE = 3.3.

The statistical studies were conducted using IBM Statistical Package for the Social Sciences (SPSS) Statistics software v28.0. The distribution of variance was tested using Levene’s test of equality. In order to evaluate the differences in colour variation values among the specimens examined in various beverages and using different polishing techniques and immersion times, a 3-way analysis of variance (ANOVA) within each staining solution was conducted including factor interaction at a significance level of α = 0.05. Subsequently, Tukey’s multiple comparisons was conducted as a post hoc test to assess and evaluate the response of each material to staining solutions.

## Results

**Figure 1:**
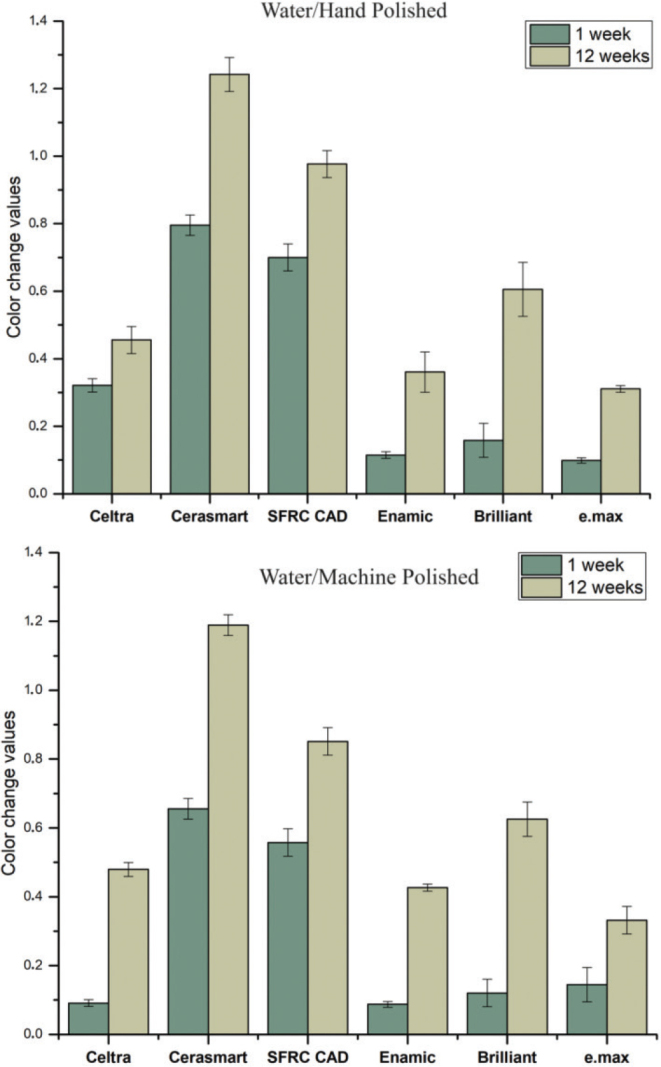
The mean colour change (∆E) values of the CAD/CAM materials measured after 1 and 12 weeks of immersion in water, considering both hand and machine polishing techniques. Polishing technique showed no significant effect (*p* > 0.05).

**Figure 2:**
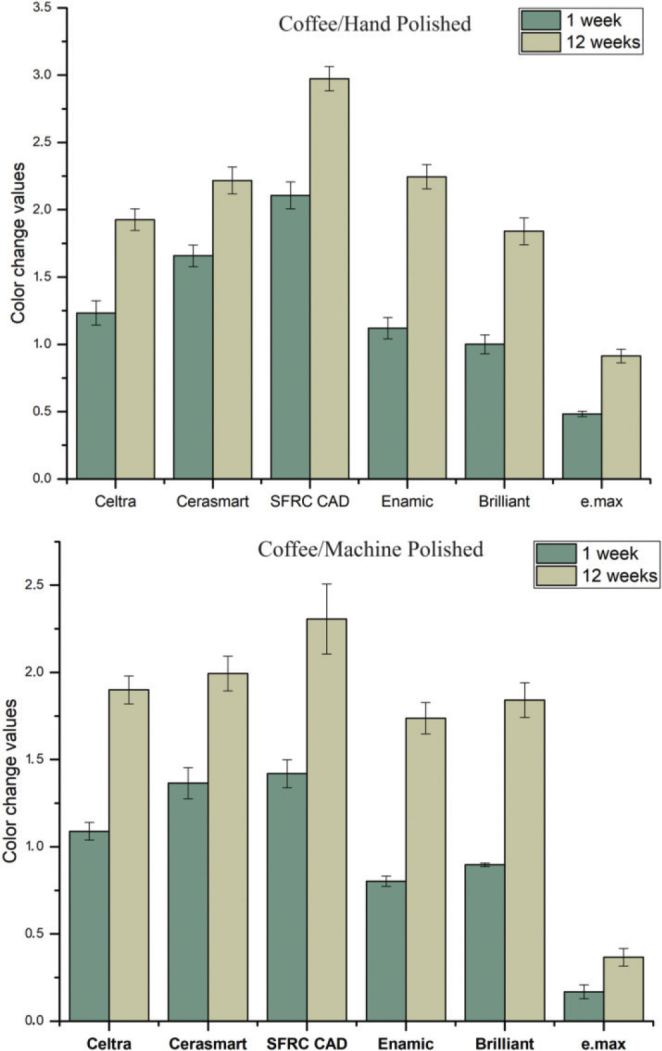
The mean colour change (∆E) values of the CAD/CAM materials measured after 1 and 12 weeks of immersion in coffee, considering both hand and machine polishing techniques.

**Figure 3:**
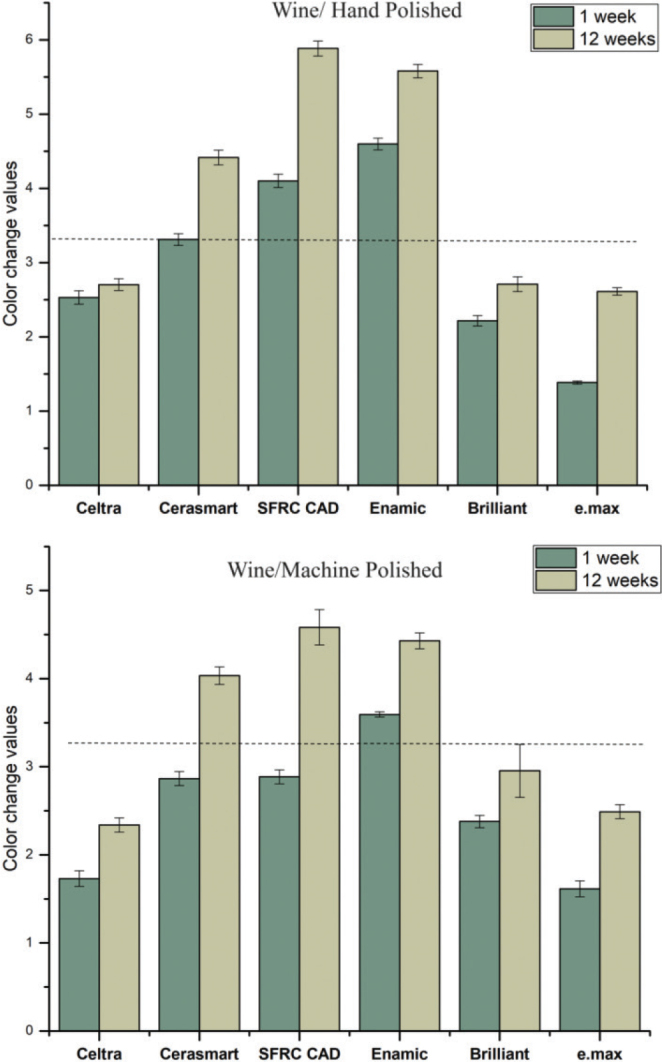
The mean colour change (∆E) values of the CAD/CAM materials measured after 1 and 12 weeks of immersion in red wine, considering both hand and machine polishing techniques. Values that are higher than the dotted line suggest clinical unacceptability, with a threshold of ∆E ≥ 3.3.

**Figure 4:**
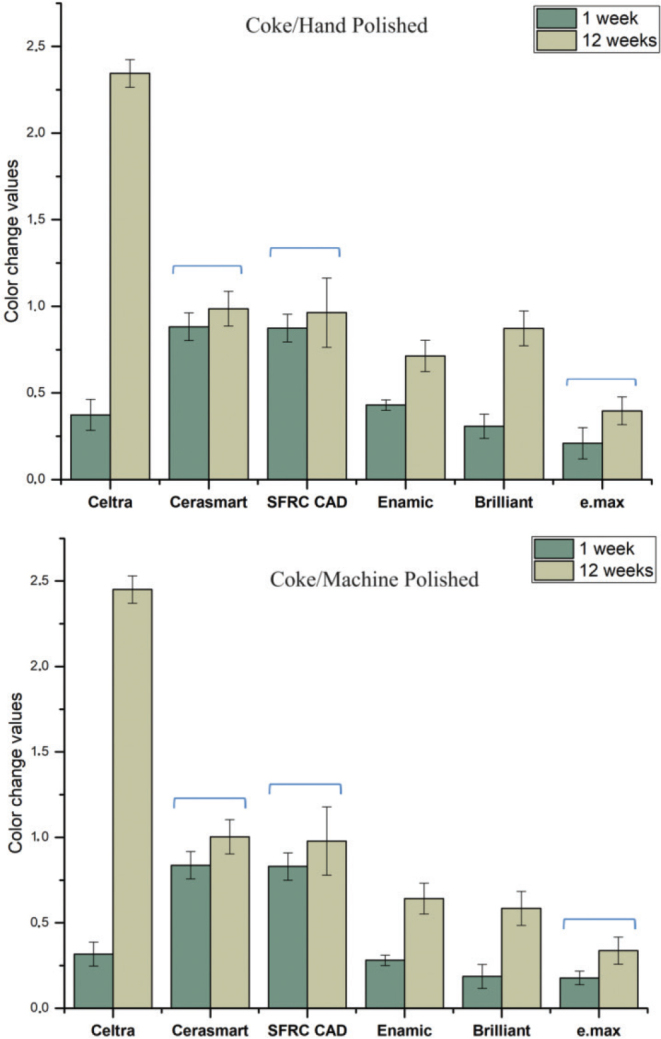
The mean colour change (∆E) values of the CAD/CAM materials measured after 1 and 12 weeks of immersion in coke, considering both hand and machine polishing techniques. Polishing technique showed no significant effect (*p* > 0.05) and groups joined by a line are not significantly difference.

Figures 1–4 illustrate the mean colour changes of specimens after 1 and 12 weeks of storage in various staining solutions, following either hand polishing or machine polishing. The material type and staining solution notably influenced the colour stability of the specimens. In each staining solution, material type, immersion time, and polishing technique demonstrated a significant effect on ∆E values (*p* < 0.05), with the exception of water and coke, where the polishing technique showed no significant effect (*p* > 0.05). Significant interaction between material type and immersion time was observed in specimens immersed in coke (*p* < 0.05).

The average ∆E values for specimens submerged in wine were determined to be substantially greater compared to the other beverages. Overall, IPS e.max exhibited the lowest ∆E values among all the tested CAD/CAM materials; however, this difference was not statistically significant (*p* > 0.05). SFRC CAD, Cerasmart 270, and Enamic displayed the highest ∆E values in wine (*p* < 0.05), indicating clinical unacceptability colour change (∆E ≥ 3.3). On the other hand, Celtra demonstrated the highest ∆E values following immersion in coke for 12 weeks (*p* < 0.05). The impact of polishing technique (hand or machine) on staining was determined to be reliant upon the specific material and staining solution employed. The utilisation of machine polishing on IPS e.max, Enamic, and SFRC CAD specimens immersed in coffee and wine resulted in notably lower ∆E values (*p* < 0.05) when compared to the hand polishing technique. Conversely, no variations were noted for other materials exposed to various staining solutions.

## Discussion

The colour stability of CAD/CAM restorations during use could be affected by the intake of diverse foods and beverages with varying pH levels, potentially causing staining and, maybe esthetic failure of the restorations [22]. This often requires replacing restorations in the aesthetic zones [23]. The phenomenon of discolouration poses a significant challenge for both patients and dentists, demanding more time and resources for retreatment.

In this laboratory-based investigation, the impact of staining solutions, immersion time, and polishing techniques on the colour stability of six distinct CAD/CAM materials were assessed, revealing statistically significant differences. The findings led to the rejection of the null hypotheses, suggesting that the colour stability of CAD/CAM restorative materials differed considerably based on the staining solution type, materials used, immersion time, and the polishing technique applied.

Based on our results, SFRC CAD revealed comparable ∆E values compared to tested composite-based CAD/CAM materials. Thereby, microfibres filler loading had no negative impact on the colour stability of the SFRC CAD. This finding aligns with prior research that showed favourable surface and optical properties of SFRC CAD when compared to various particulate-filled composites [11, 14, 16]. Within the mentioned research, the microfibres of the SFRC CAD did not protrude from surface after polishing or wear test; instead, they were polished in the resin matrix, resulting in often a uniformly smooth surface. Our results align with the study conducted by Uctasli et al., where flowable SFRC (everX Flow) demonstrated colour stability comparable to other conventional particulate-filled composites that were tested [18]. These results give an indication that SFRC CAD has the potential to be utilised safely as monolithic restorations across a wider variety of clinical scenarios.

Generally, the colour stability of composite-based or hybrid CAD/CAM materials was inferior in comparison to glass-ceramic materials. The staining susceptibility of CAD/CAM composites can be affected by different parameters, including the extent of water absorption and the hydrophilicity of the composite matrix [24]. If a composite material is hydrophilic, it is also susceptible to absorbing various fluids, which might lead to discolouration. The diverse compositions of composites result in variations in their sensitivity to staining, principally due to their water absorption characteristics. Water is thought to act as a channel for stains to enter the composite matrix [25]. The particulate fillers in CAD/CAM composites do not take in water throughout the material, although they can absorb water on their surface. A higher amount of resin matrix in the composites results in higher water absorption and diminished bonding between the resin matrix and filler particles. Swelling and plasticising actions can cause the production of microcracks in the composite matrix, along with the development of gaps between the filler and matrix. These conditions promote the entry of stains and the resulting colour change of the restorations [25].

It is well acknowledged that glass-ceramic CAD/CAM materials have better colour stability than composite-based CAD/CAM materials [26]. This is in line with our results, as IPS e.max showed better colour stability than composite-based CAD/CAM materials. While Celtra is a glass-ceramic material, its colour stability was found to be lower than that of IPS e.max. The colour stability of glass-ceramic materials can be influenced by several parameters, such as the crystalline structure, grain size, and porosity [22].

Discolouration can be evaluated by either visual or instrumental methods. We utilised spectrophotometry in our investigation, a method that eliminates any subjective interpretation when doing visual colour comparisons. The reliability of spectrophotometry as a tool in dental materials studies has been acknowledged [18, 27]. The ∆E value quantifies the extent of colour changes that an observer may see in the materials either after immersion or over a period of time. Thus, ∆E is a more significant measure than the separate ∆L, ∆a, and ∆b values [28].

It is crucial to note that within dental research, two colour difference formulas have been utilised extensively over the years [29]. Recent research has indicated that the CIEDE2000 colour difference formula, compared to the CIE LAB formula utilised in our study, offers a superior fit when assessing visual tolerances [29–32]. The CIEDE2000 formula is believed to more accurately reflect human perception of colour differences, with a reported 95% agreement with visual observations, compared to the 75% agreement of the CIE LAB formula [30, 31]. However, CIE LAB formula remains applicable and valid for calculating colour or translucency differences across materials [32].

We evaluated the colour stability of CAD/CAM materials by submerging them in four frequently consumed beverages (water, coffee, red wine, and coke) over a period of 12 weeks. In order to mimic real patterns of beverage consumption, we took into account the mean duration for consuming a cup of coffee as an example, which is roughly 15 min. Furthermore, taking into account the typical coffee intake of three cups per day among consumers of coffee, our 12-week immersion period accurately replicated the experience of consuming the beverage for a duration of 7 years [33].

Our investigation validated prior research findings [34, 35] that red wine causes the most noticeable discolouration of CAD/CAM materials, which is easily visible without the need for a high level of inspection. The discolouration caused by coffee and wine can be explained by the absorption of their pigments into the organic component of the composites [33]. Remarkably, coke containing phosphoric and citric acids, with a pH of around 2.5, did not exhibit a significant correlation with changes in colour in CAD/CAM materials when compared to coffee and wine. Acids can exhibit different behaviours in terms of facilitating dissolution, absorption of water, and erosion of materials. Bagheri et al. and Ertas et al. have proposed that the lack of yellow colourants in coke leads to reduced discolouration compared to the staining induced by coffee and wine [25, 36]. In addition, numerous studies have demonstrated that, despite their acidic nature, carbonated beverages like coke cause only modest discolouration of CAD/CAM materials when compared to other dark beverages [26, 35].

According to Paravina and his colleagues, the colour difference threshold for teeth and tooth-coloured restorations was noted to be 2.7 when utilising the CIE LAB formula [30]. However, various other studies have reported divergent values, a difference attributed to variations in the number and types of observers, research locations, and the colour difference formulas employed [32]. Multiple investigations have indicated that ∆E values ranging from 1 to 3 can be detected by the eye alone; however, ∆E levels beyond 3.3 are thought to be clinically unacceptable [18, 20, 21]. Based on these principles, the CAD/CAM materials examined in this work showed satisfactory colour stability when subjected to staining solutions for a duration of 12 weeks, except when exposed to wine. Among the tested materials, only SFRC CAD, Cerasmart 270, and Enamic in wine exhibited colour change values surpassing 3.3 (Figure 3). The inclusion of UDMA in the structure of these CAD/CAM materials (Table 1) may explain this behaviour. These materials are prone to releasing monomers in acidic conditions more than Brilliant, which is formulated with Bis-GMA and Bis-EMA resin.

The differences in colour stability reported among the investigated composite-based CAD/CAM materials might also be related to the variations in filler/resin ratio (Table 1). Furthermore, the colour change that was noticed might also be linked to the utilisation of the silane agent. The discolouration caused by silanisation of inorganic particles is attributed to the silane’s high water sorption capacity [37]. Moreover, the shade of the material is an additional factor that affects CAD/CAM composite staining. The inclusion of pigments in darker colours typically enhances their ability to achieve accurate colour matching. Consequently, translucent shades, which do not include these pigments, may undergo a more pronounced level of colour change [38].

The literature has identified polishing methods as a potential element that can impact colour stability. Nevertheless, there is a lack of agreement regarding the influence of surface treatment on the change of colour caused by staining. Conflicting reports exist about the results of different polishing methods [39–42]. Several research proposes that the level of smoothness in a surface does not always result in higher resistance to staining. Conversely, other studies indicate that the lack of appropriate polishing can make specimens more prone to staining. The current research on the correlation between surface treatment and colour stability remains unclear, necessitating additional investigation to provide clarity on this issue. For this investigation, the Sof-Lex™ Polishing System was used to polish one surface of all the CAD/CAM specimens, aiming at accurately imitating practical situations in clinical settings. The opposite side of the specimens was subjected to a precise laboratory method including 4000-grit polishing, resulting in a highly polished surface. Surprisingly, the results demonstrated that the polishing procedure did not exert a notable influence on the colour change of the composite specimens across all CAD/CAM materials. This result aligns with the investigation conducted by Imamura et al., which also demonstrated that the polishing technique had a restricted influence on the discolouration of composites [37]. It is also in line with the research conducted by Sasany et al., who illustrated the successful use of the Sof-Lex™ polishing system for chair-side CAD/CAM material polishing [40].

Research evaluating colour stability in vitro has inherent methodological limitations. The aim of this study was to mimic the prolonged impact of oral environmental factors within a condensed 12-week timeframe, with the goal of predicting the colour stability of the SFRC CAD material. It is crucial to acknowledge that in the oral environment, elements such as heating from food and drink, saliva, and other fluids can exert a more substantial impact. In addition, chewing can modify the texture of the composite material, resulting in the retention of deposits and elements that cause discolouration on uneven surfaces for extended durations.

## Conclusions

The following conclusions can be drawn, given the limitations of the present study:

1. The colour stability of tested SFRC CAD, was comparable to other composite-based CAD/CAM materials.
2. The extent of colour change was influenced by the staining solution, immersion time, polishing technique, and the specific material used.
3. Among all of the tested CAD/CAM materials, IPS e.max showed the highest level of colour stability.

## Declarations

### Ethics approval and consent to participate

Not applicable.

### Consent for publication

Not applicable.

### Availability of data and materials

The datasets used and/or analysed during the current study available from the corresponding author on reasonable request.

### Conflicts of Interests

Author P.V. consults for Stick Tech – Member of the GC Group in R&D and training. Other authors declare to have no conflict of interests.

### Authors’ contributions

Study design, L.L. and S.G.; collection of data, S.G.; and U.M.B. and K.W.; data analysis/interpretation, S.G., U.M.B. and L.L.; writing-original draft preparation, S.G.; writing-review and editing, U.M.B, L.L. and P.V.
